# Methadone for Analgesia in Children with Life-Limiting Illness: Experience from a Tertiary Children’s Health Service

**DOI:** 10.3390/children5070086

**Published:** 2018-06-27

**Authors:** Christine Mott, Amrita Sarpal, Krista Moss, Anthony Herbert

**Affiliations:** 1 Lady Cilento Children’s Hospital, Brisbane 4069, Australia; Anthony.herbert@health.qld.gov.au; 2 Children’s Hospital London Health Sciences Centre, London, ON N6A 5W9, Canada; amrita.sarpal@lhsc.on.ca; 3 Alder Hey Children’s Hospital, Liverpool L12 2AP, UK; mos.krista@gmail.com; 4 Centre for Children’s Health Research at Institute of Health and Biomedical Innovation, Queensland University of Technology, Brisbane 4069, Australia

**Keywords:** methadone, analgesia, life-limiting, children, paediatric, opioid

## Abstract

Methadone has the potential to assist in the management of pain in children with life-limiting illness, but its use is limited by its complex pharmacokinetic profile and limited research on its use in children. This is a retrospective review of the use of methadone as an analgesic in 16 children with life-limiting illness. Efficacy, dosing and side effect profile were analysed. Fifteen (94%) patients had improvements in their analgesia with minimal observed adverse effects. Patients were either rapidly converted from a prior opioid in one change or received methadone as an adjunct medication. Conversions were calculated using ratios frequently in the range of 10:1 to 20:1 from the oral morphine equivalent total daily dose (MEDD). Adjunct initial dosing was a low dose trial, often beginning with 1 mg at night. Only two patients required a dose adjustment due to side effects attributed to methadone. This was despite the cohort having significant underlying illnesses, extensive concurrent medications, and high methadone dosing where needed. Analysis of dosing and ratios indicates that an individualised approach is required. Based on this and on the infrequency of methadone use in this population, specialist assistance with dosing is recommended. Further research, including prospective and pharmacokinetic studies, is recommended.

## 1. Introduction

Methadone is a long-acting lipid soluble synthetic opioid analgesic frequently used in adult palliative care, and increasingly in paediatrics. Methadone is generally used when other opioid analgesic agents have failed to achieve sufficient analgesia or are causing excessive side effects. Due to the presence of inactive metabolites with methadone it is well tolerated in renal failure and avoids the anti-analgesic or potentially hyper-analgesic metabolites seen with morphine [1,2]. Efficacy is also maintained in stable liver disease [2].

Methadone acts primarily at mu receptors but also serotonin, noradrenaline and *N*-Methyl-D-aspartic acid (NMDA) receptors. Consequently, it may be associated with less sedation and development of tolerance [3,4,5]. It is also a lower cost option compared to other opioids in the American and Australian contexts [2]. Routes of administration can include oral, rectal, nasal, sublingual, subcutaneous, epidural or intravenous [6]. The half-life of methadone varies significantly between individuals and over time, and is shorter in children, [7,8,9]. With chronic administration the half-life in adults drops from 54.8 h to 24 h [10]. It can take 4 to 10 days to achieve a steady state, and most deaths attributed to methadone overdose occur in the first 4 to 6 days of commencing treatment due to this potential for accumulation [2].

Methadone has the potential for significant respiratory depression and prolongation of the interval between the start of the Q wave and the end of the T wave (QT interval). Methadone pharmacokinetics can also be altered by concurrent use of medications that access CYP3P4A or CYPP2B6. This includes corticosteroids, fluconazole, erythromycin, ketoconazole, amitriptyline, carbamazepine and phenytoin [2,11]. There are also social barriers to the use of methadone, including stigmatization related to its use in addiction medicine [12,13].

Methadone is the oral analgesic of choice in Finland for paediatric palliative care, and is used in paediatric persistent pain in Israel, however the literature base in paediatric populations is limited [14,15]. Case series have shown methadone to be effective in paediatric pain management related to surgery, cancer and sickle cell disease [3,11,12,15,16]. In paediatric oncology patients, it has been found to be beneficial in managing vincristine-induced neuropathy and at end of life [11,16]. An important study by Davies et al. found pain management was improved by parental report in 16 of 17 children aged 2 to 18 years with advanced cancer when trialed on methadone [12]. Another study in oncology patients found methadone was effective for analgesia in 64.3% of participants of average age 15.7 years (range 0.6–23) [16]. It is also helpful in the management and prevention of opioid withdrawal in children [16].

One-off or initial dosing used in paediatric studies generally ranged from 0.15 to 0.3 mg/kg, with one study reporting an intravenous loading dose of 0.6 mg/kg [7,17,18]. Another study suggested a starting dose of 0.1 mg/kg every 6 to 8 h for opioid-naïve patients [15]. The largest initial dose reported in an opioid-naïve patient was 20 mg [18].

Compared to other opioids where a simple ratio tool can be applied, methadone requires a graded conversion ratio based on prior opioid exposure, due to its complex pharmacokinetics [2,12,19,20]. A low opioid exposure (low oral morphine equivalent total daily dose (MEDD) and shorter duration of use) requires a relatively higher methadone conversion ratio, while a high exposure requires a comparatively lower methadone conversion ratio. Therefore, a ratio of 1:1 for conversion from morphine may be appropriate for an opioid-naïve patient but may lead to significant adverse effects where high-dose opioids have been used prior [2]. A morphine-to-methadone conversion ratio of 20:1 has been suggested in both adults and children receiving more than 1000 mg of daily oral morphine [2,20]. A ratio of 10:1 has been suggested if the daily oral dose of morphine is less than 1000 mg [12]. Some studies suggest that the maximum daily dose a patient should be commenced on is between 50 and 60 mg of oral methadone [19,21].

There are three common strategies which have been described when commencing an opioid rotation to methadone: ‘rapid conversion’, ‘three-day switch’ and ‘ad libitum’ [2,22]. A ‘rapid conversion’ involves stopping the prior opioid completely while a conversion ratio guides replacement methadone dosing. A ‘three-day switch’ involves titration of methadone dosing to effect and concurrent decrease of the prior opioids over three days. Finally, the ‘ad libitum’ method uses the amount and frequency of as-needed doses as a guide to subsequent regular dosing. Use of ‘ad libitum’ strategies in children can be complicated by a patient’s inability to directly request medication due to developmental limitations, and a need to observe standard inpatient restricted drug protocols. A systematic review of these methods found rapid conversion to be the least effective and most likely to cause adverse effects [22]. A previous paediatric study found similar success with both rapid and titrated conversion “over a few days” [12]. Other systematic reviews have failed to demonstrate evidence of superiority of any one method [23].

Side effects of methadone in children in prior studies have been minor in both oncology and post-operative settings. Adverse effects have included somnolence, mild respiratory depression, hallucinations, nausea and constipation [12,17]. With one-time doses, sixty-five children undergoing operations had no major adverse events [8,18]. With longer duration of dosing, for mixed indications, sedation was noticeable in approximately one quarter of patients in one study of 41 children [16]. With higher doses of methadone (25 to 33 mg/kg/day) adverse events have included drowsiness, hypotension, hypoventilation and central nervous system depression [11,18]. Chronic methadone therapy for analgesia contributed to the development of severe obstructive sleep apnoea in one case study [24]. A specific concern with methadone use in all populations has been the risk of changes in cardiac physiology. Reversible alterations in QT interval have been noted in children receiving methadone, with concurrent use of medications such as fluconazole [11]. Changes in QT interval length have not reached a point of clinical relevance in paediatric studies, however, even in neonates and with long duration of use [7,11,25]. Despite this, and recent changes to the intravenous formulation that further reduce risk, it is recommended that family history is considered before initiation [26,27].

The current study reviews children with life-limiting illnesses (cancer and non-cancer) prescribed methadone for analgesia, including at end of life. The outcomes of interest were efficacy for analgesia, side effect profile and dosing regimens to achieve analgesia. This study aims to contribute to the growing literature on the use of methadone for analgesia in paediatric patients with life-limiting illness.

## 2. Methods

All patients prescribed methadone for analgesia were identified from the palliative care database covering one tertiary children’s health service in Brisbane, based at the Royal Children’s Hospital from 2008 to 2014, and the Lady Cilento Children’s Hospital from 2014 to 2015, after the former ceased operations. The focus of the study was children with both complex pain and serious, life-limiting illness. Patients given methadone exclusively to assist with weaning opioids following Paediatric Intensive Care Unit (PICU) admission, and not for analgesia, were excluded from the audit. A retrospective chart audit was undertaken for the 11 patients identified through the database. Five additional cases eligible for inclusion in the study were identified prospectively, resulting in a total of 16 patients included in the audit. The current study was approved by Children’s Health Queensland Human Research and Ethics Committee (HREC/12/QRCH/11, 12 January 2012).

Demographic data identified included the patient’s age, sex and underlying diagnosis. Pain characteristics and prior treatments were documented, including the analgesic regimen administered immediately prior to commencing methadone. Assessments of pain severity were based on documented parental report, clinician review and self-report, as appropriate. Documented regular medications given concurrently with methadone and side effects attributed to methadone were also reviewed.

An equivalent total daily dose of oral morphine (MEDD) was calculated for each patient to allow easier comparison and analysis. A paediatric source [28] was used to advise best equivalency, supplemented with other sources where needed [29]. An oral-to-intravenous ratio of 3:1 was used for morphine, and a conservative 2:1 oral-to-intravenous ratio of was used for methadone. The MEDD was calculated based on the total scheduled dosing of prior opioids received in 24 h only, breakthrough dosing was not included in this calculation. Table 1 shows the opioid equivalencies used to calculate the MEDD [29].

## 3. Results

Sixteen children were administered methadone over an 8-year period for analgesia in the context of various life-limiting illnesses. Demographic details are listed in Table 2. Most patients had cancer (12 of 16, 75%), 3 had neurological diagnoses (19%) and 1 had a cardiac diagnosis (6%). There was an even distribution of male and female patients. Patients ranged in age from 1.3 to 16 years (mean 7.4 years).

All patients had either overt reported pain or symptoms interpreted by parents and clinicians to be indicative of pain. In six (38%) of the patients, pain was determined by proxy because the patient was either developmentally incapable (through impairment or age) of verbal expression or too unwell to communicate. Findings documented at review of children with pain most commonly mentioned generalised distress (69%), variably documented as “distress”, “irritability”, “agitation” or being “unsettled”. Other common findings associated with pain were decreased mobility (19%) and decreased sleep (19%), with some patients displaying more than one of these features. 

Nine patients received methadone for pain management (where goals of care still included disease modification or cure). Seven patients received methadone in a palliative care context (where goals of care were primarily comfort-focused, often at end of life) (See Table 2). All patients had methadone commenced by the same consultant who specialised in palliative care, and also regularly managed non-palliative patients with cancer and non-cancer pain.

The MEDD used at the time of commencing methadone, and the methadone dose (initial and highest) are displayed with the demographic details in Table 3, illustrating the frequency of high opioid dosing prior to methadone administration. Methadone was found to be generally well tolerated and safe. Of the 16 patients reviewed, 15 (94%) continued the medication and had improved outcomes determined either through improved pain or reduced requirement of other analgesia such as breakthrough medications. One patient discontinued methadone due to perceived lack of effect.

Adverse symptoms in the early phase of treatment, summarised in Table 3, included drowsiness, unsteadiness, itch, constipation, transient bradypnoea and behavioural change. One patient experienced a seizure, for which methadone was considered as one of several potential pharmacological causes, in the setting of complex multi-system disease. Three patients experienced signs of withdrawal, including rebound pain and labile emotions, when methadone dosing or absorption was reduced. In two patients, the dose of methadone was reduced due to adverse symptoms attributed to methadone (unsteadiness and a minor head injury caused by a fall).

Of the patients reviewed, nine patients have subsequently died. All deaths were expected. Four of these patients died within 16 days of commencing methadone, and three of those patients had been on high-dose opioids prior to commencing methadone, using the previously established definition of high dosing as dosing above 3 mg/kg/h of intravenous morphine equivalent [30]. Five patients showed clinical improvement or stabilisation of their symptoms and were subsequently successfully weaned off methadone. For two patients, methadone has been weaned without cessation at completion of review.

Medications administered concurrently when methadone was commenced were reviewed as part of this study. It was noted that in several cases these medications included those medications with the potential to cause QTc prolongation [31]. Not all patients had an electrocardiogram (ECG) prior to commencement, particularly where the patient was at end of life. The list of concurrent medications included alternate analgesic medications, antibiotics, anti-seizure medications, anti-emetics, steroids and other targeted treatments for underlying conditions. Planned concurrent chemotherapy agents were not considered. Many of the patients required extensive concurrent medications for optimal management, due to severity of illness or symptomatology.

Two cohorts emerged within the group of children analysed. One group of children were given methadone as a complete replacement for other opioids (*n* = 12). This occurred in a situation where high-dose opioids had preceded the change to methadone (see Table 3). Another group of children were given methadone as an adjunct analgesic for difficult-to-manage pain (*n* = 3). This latter group of children often subsequently transitioned to methadone as a primary opioid over days or weeks. One child commenced methadone de novo, the only occasion where dosing was wholly based on patient weight.

The most common reason for a change to methadone (*n* = 9) was incomplete analgesia while on other opioids. This was often morphine or hydromorphone given at a high dose. Other reasons for rotation to methadone included side effects from other opioids (*n* = 6) and a desire to use oral analgesic medications (*n* = 3). For some patients, the change to methadone was multi-factorial. 

**Figure 1:**
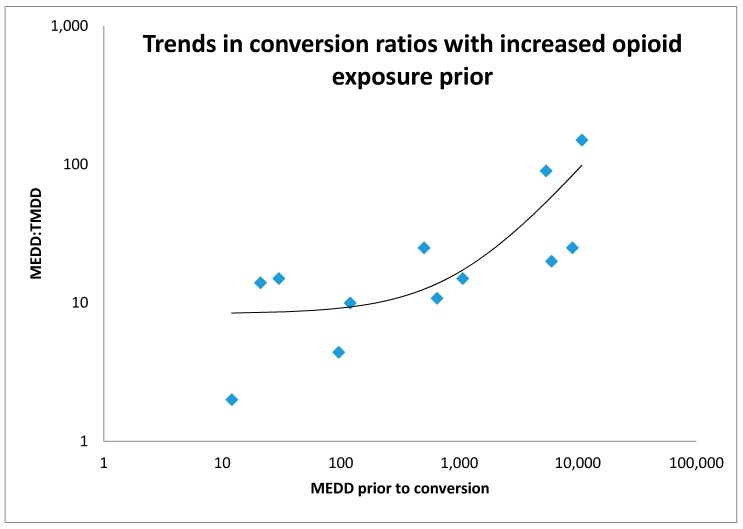
Logarithmic curve of methadone conversion ratios used for increasing MEDD prior to conversion. Only rapid conversion ratios considered for this graph.

Routes of administration included enteral (*n* = 8), sublingual (*n* = 2), intravenous (*n* = 4) and subcutaneous (*n* = 2). Twelve children were rapidly converted from a prior opioid to methadone and conversion ratios from equivalent daily oral morphine equivalent ranged from 2:1 to 150:1. Half of the conversions to methadone were in the range 10:1 to 20:1, two received less than 10:1, and four received greater than 20:1. The lowest conversion ratio of 150:1 correlates with the highest equivalent prior MEDD (10,800 mg) of the patients analysed. This patient subsequently needed incremental dosing up to 600 mg daily of infused subcutaneous methadone, representing a conversion of 13:1 from the original morphine equivalent, in the context of end-of-life care. A graph of conversion ratio to MEDD prior to conversion is provided with an average line on a logarithmic scale (Figure 1), demonstrating an expected increase in ratio used with increased previous opioid exposure.

Where methadone was used as an adjuvant (*n* = 3), the starting dose was 1 mg at night intravenously or sublingually, in addition to the prior opioid used. These patients subsequently rotated to methadone as their regular opioid analgesic over a period of days to weeks.

Analgesic strategies immediately prior to the introduction of methadone were most commonly hydromorphone infusions (10 patients), concurrent ketamine infusion with opioid infusion (six patients), concurrent benzodiazepines with opioid management (five patients), neuropathic agents (gabapentin, pregabalin or amitriptyline) (four patients), fentanyl infusion (two patients), oral opioids (codeine, morphine or oxycodone in three patients), morphine infusion (one patient) and dexmedetomidine infusion (one patient in PICU). The majority (56%) of patients were also accessing documented non-pharmacological strategies. The most common was music therapy (*n* = 5), followed by occupational therapy, physiotherapy, parent-led distraction, and acupuncture.

## 4. Discussion

Increasing the range of safe and effective methods of providing analgesia in children is important, particularly in life-limiting illness and at end of life. Methadone was infrequently used at the sites involved in this study but was effective for analgesia and had a tolerable side effect profile in the majority of cases reviewed. All patients in this study had alternate opioid medications for analgesia, and many were on high-dose opioids, prior to commencing methadone. The majority had been on an alternate opioid and a sedative infusion (midazolam, ketamine or dexmedetomidine) prior to starting methadone. Methadone was effective even when children had been on significant analgesia and sedation, sometimes requiring PICU support, prior to its commencement. These findings and low subject numbers overall may indicate that methadone was a ‘last resort’ in analgesia in the Queensland context.

In contrast to findings in one systematic review, the current study demonstrated tolerability and efficacy of rapid conversion techniques [22]. In the current study, rapid conversion was indicated for individual treatment needs, such as pain crisis or technical inability to continue increasing other measures. It is possible some of the initial conversion ratios and nocte dosing of methadone in the current audit were conceived as a longer-term conversion strategy with subsequent titration of dosing expected. The current audit still assists in defining safe initial dosing with prior opioid exposure though.

**Figure 2:**
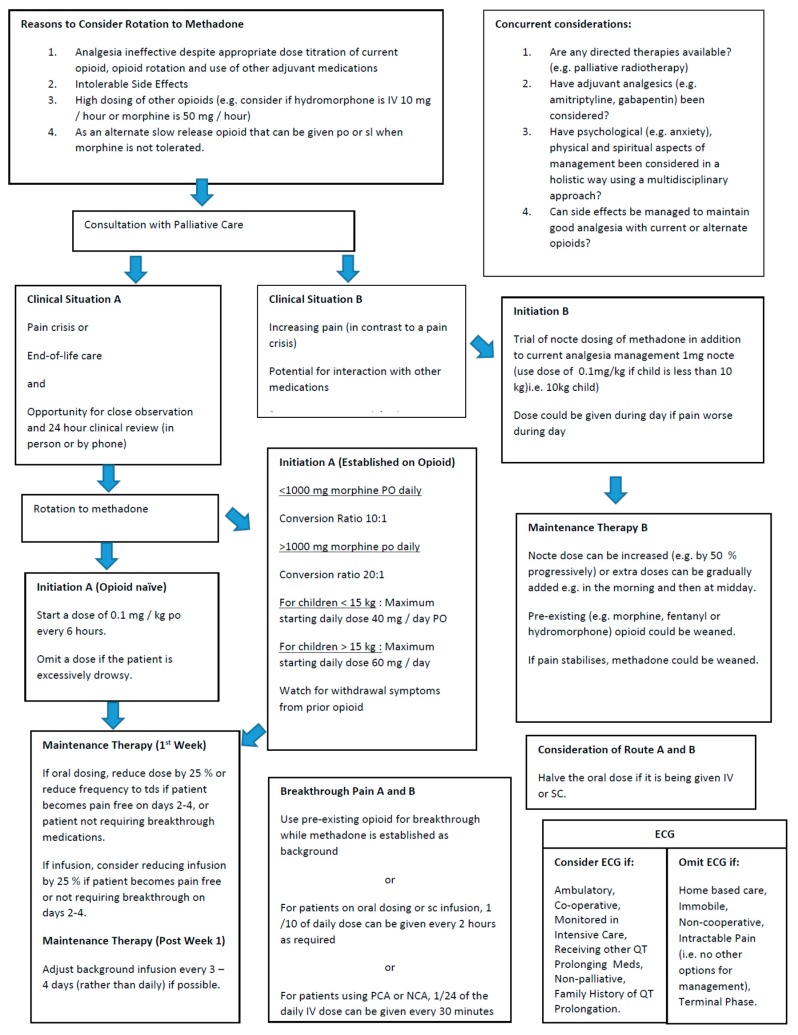
A suggested guideline in considering methadone administration to manage pain in children.

Review of dosing practices within this study shows that individualisation is needed. Figure 2 demonstrates the various approaches to commencing methadone used in the current study, and is a guide based on our review of these cases and relevant literature. Where possible, we suggest that a practitioner with experience in prescribing methadone is involved in the initiation of methadone as an analgesic, and due care is exercised when initiating or rotating to methadone. Individual patient factors such as goals of care, care setting and previous type and dose of opioids used need to be considered in this process. Rotation to methadone should be seen as a “care pathway” rather than a “dose calculation”.

The trend with commencement of methadone was either to add methadone to existing analgesic regimens as an adjuvant, or to rapidly convert current opioids to methadone. This would also include the discontinuation of ketamine. The ratios used to calculate an appropriate starting dose of methadone varied, with ratios closer to 1:1 used for lower prior opioid exposure, and ratios around 20:1 to 25:1 where there had been higher prior opioid exposure. This is appropriate given the literature on methadone’s complex pharmacokinetics. Where methadone was used as an adjuvant treatment, dosing was generally 1 mg at night intravenously or orally. Based on the current audit, a suggested guide to initial dosing is provided in Figure 2. Due to its prolonged half-life, dose titration after commencing methadone should occur every three days where possible, with careful review and consideration of side effects such as drowsiness.

Methadone administration route in this study was primarily related to patient practicalities, for example, oral route for ongoing use with stable-phase disease and subcutaneous use at end of life. Dosing intervals for oral therapy should be shorter than with adult patients based on difference in pharmacokinetics and may need to become shorter over time [9,10]. Due to the challenges of individual pharmacokinetics and a tendency to initially under-dose methadone [32], careful observation and appropriate titration of methadone may be necessary. For this reason, it is often recommended that patients be hospitalised during initial therapy. Research has found inpatient titration is faster [21]. In Davies’ study, 6 paediatric patients commenced methadone in the community, where there was perceived parental reliability and provision for extra in-home monitoring including regular phone calls. Only one of these patients subsequently required related admission for monitoring [12]. The specific dosing used in this setting was not provided. Studies in adult patients found outpatient titration to be safe and effective [21]. In the current audit, 75% of patients (*n* = 12) commenced methadone as an inpatient. However, inpatient admission in our context may be related to concurrent ongoing treatments and remaining at home may be related to the goals of the child and family during end-of-life care. If methadone is to be commenced at home, the parents need to be reliable in their ability to report pain and side effects, and provision of significant in-home support must be possible.

Due to significant poly-pharmacy related to complex underlying conditions and symptoms, side effects patients experienced were possibly multi-factorial. Milder side effects possibly were not documented in the context of severe underlying illness and related symptomatology. It is also important to note that six of the patients had side effects from previous opioids significant enough to require opioid conversion to methadone, whereas 15 of 16 patients then found methadone tolerable. Although significant cardiac events were not noted with children in this study (despite three patients receiving greater than 200 mg per day dosing, which is thought to offer significant electrophysiologic risk [2]), consideration of whether to perform an ECG remains important and recommendations feature in the guideline (Figure 2).

This retrospective audit was limited by the quality and quantity of documentation taken for each child, although this was augmented by having the prescribing clinician review documentation as well. Retrospective chart analysis with an outcome of “negative” or “positive” tone in records after starting an intervention has previously been used in both paediatric methadone research and in other medication research in paediatric palliative care [12,33]. Ideally a pre- and post-intervention pain score should be used in future prospective analysis.

A recent review examined necessary dose alterations after commencing methadone in children, to assess efficacy of initial dosing ratios used [34]. An issue with this approach is that at end of life escalating pain is known to occur, and so necessary increments in dosing after commencement could be due to initial under-dosing or may represent appropriate increases related to disease progression. For these reasons, separate consideration of efficacy from initial dosing ratios was not undertaken in the current study but would be an area of interest for future audits.

Three patients (19%) receiving end-of-life care with an underlying oncology diagnosis died within 7 days of commencing methadone. Previous studies have found use of opioids in palliative care does not hasten death; these three deaths were attributed wholly to underlying disease processes [35,36,37].

## 5. Conclusions

Methadone contributed to effective analgesia in most paediatric patients with complex cancer and non-cancer life-limiting diagnoses (94%; 15 of 16 patients). Methadone was generally well tolerated despite significant disease burden and extensive concurrent medications. Methadone has the potential to offer a promising alternative to other opioids in paediatrics. A guideline with suggested dosing conversions from morphine equivalent is provided based on prior opioid exposure. This audit will give clinicians greater confidence in prescribing methadone, particularly in terms of dosing regimen. Greater confidence may lead to initiation of methadone earlier in the disease continuum when appropriate, rather than it being used as a medication of “last resort”. Ongoing research is required to assist in developing appropriate recommendations and guidelines for use of methadone as an analgesic in children with life-limiting illness. 

## Figures and Tables

**Table 1:** Conversion ratios used (parenteral form).

	Morphine	Fentanyl	Hydromorphone
Relative Potency	1	40	5
Example Doses	4 mg	100 mcg	800 mcg

**Table 2:** Patient demographics.

Patient	Age	Sex	Weight (kg)	Diagnosis	Prior Opioid/Analgesia	Care Location	Goals of Care	ECG
1	4 years	F	19.0	Relapsed biliary embryonal rhabdomyosarcoma	Hydromorphone	Hospital (ward)	Palliative Care	Yes
2	2 years	M	12.7	Haemophagocytic lymphohistiocytosis	Hydromorphone, ketamine	Hospital (ward)	Pain Management	No
3	8 years	F	27.0	Relapsed acute lymphoblastic leukaemia	Hydromorphone, ketamine	Hospital (intensive care)	Palliative Care	Yes
4	3 years	M	14.5	Metastatic hepatoblastoma	Hydromorphone	Hospital (ward)	Pain Management	No
5	5 years	M	21.0	Relapsed acute lymphoblastic leukaemia	Hydromorphone	Home	Palliative Care	No
6	16 months	M	11.0	Meningococcal septicaemia with brain injury and four limb amputation	Morphine	Hospital (ward)	Pain Management	No
7	12 years	M	34.6	Relapsed pelvic alveolar rhabdomyosarcoma	Hydromorphone	Home	Palliative Care	No
8	9 years	F	45.9	Frontotemporal pleomorphic xanthoastrocytoma	Hydromorphone	Hospital (ward)	Pain Management	No
9	16 years	M	46.0	Refractory graft versus host disease post bone marrow transplant for acute myeloid leukaemia	Hydromorphone	Hospital (ward)	Palliative Care	No
10	16 years	M	38.6	Cerebral palsy with spastic quadriplegia, parental nutrition	Fentanyl patch	Hospital (ward)	Palliative Care	No
11	6 years	F	20.0	Low grade sarcoma	Hydromorphone	Hospital (ward)	Pain Management	No
12	5 years	F	18.0	Juvenile myelomonocytic leukaemia, graft versus host disease	Hydromorphone, ketamine	Hospital (intensive care)	Pain Management	Yes
13	7 years	F	25.8	Epileptic encephalopathy	Oxycodone	Home	Pain Management	No
14	7 years	F	22.0	Pulmonary atresia with ventricular septal defect and major aortopulmonary collateral arteries	Morphine	Home	Palliative Care	No
15	15 years	F	45.0	Severe veno-occlusive disease and renal impairment post bone marrow transplant for acute lymphoblastic leukaemia	Hydromorphone	Hospital (intensive care)	Pain Management	Yes
16	23 months	M	15.0	Bi-lineage leukaemia requiring bone marrow transplant	Hydromorphone	Hospital (intensive care)	Pain Management	Yes

ECG: refers to whether a recent ECG (electrocardiogram) was available at the time of commencing methadone.

**Table 3:** Summary of methadone usage.

Pt	Approx. MEDD (PO)	Methadone Starting Dose and Route	Maximal Methadone Dose and Route	Rapid Conversion or Adjuvant	Reason for Rotation to Methadone	Conversion Ratio (PO Morph: PO Meth)	Breakthrough Analgesia	Side Effects or Issues Noted	Outcome
1	504 mg	5 mg QID PO	15 mg PO QID.	Rapid conversion	Increasing Drowsiness and Inadequate Analgesia.	25:1	Hydromorph PCA IV.	Less drowsy on methadone. Able to start walking again. Withdrawal symptoms.	Died 6 months after commencing methadone.
			Changed to 30 mg IV infusion over 24 h				Changed to meth PCA IV subsequently		
2	1080 mg	1 mg nocte NG	2 mg QID SL	Adjuvant	Loss of central venous access and unable to maintain SC route.	N/A	Hydromorph PCA and ketamine IV	Drowsiness and low respiratory rate requiring doses to be skipped.	Died 3 months after commencing methadone.
3	5400 mg	15 mg QID PO	240 mg IV infusion over 24 h	Rapid conversion	Inadequate Analgesia.	90:1	Hydromorph PCA IV.Eventually converted to meth PCA IV	Improved Analgesia.	Died one month after commencing methadone.
4	12 mg	1.5 mg QID PO	3 mg TDS PO. Changed to Fentanyl patch	Rapid conversion	Side effects with morphine—inadequate analgesia and itch. Methadone only alternative slow release opioid that comes as elixir.	2:1	Hydromorph NCA	Unsteadiness attributed to methadone.	Died 3 months after ceasing methadone.
5	6000 mg	150 mg SC infusion over 24 h	150 mg SC infusion over 24 h	Rapid conversion	Inadequate Analgesia.	20:1	Meth SC	Improved analgesia.	Died 7 days after commencing methadone.
6	96 mg	5.5 mg QID NG	5.5 mg QID NG	Rapid conversion	Irritability.	4.4:1	Morph NCA IV	Withdrawal symptoms.	Weaned off methadone. Alive.
7	9000 mg	180 mg SC infusion over 24 h	324 mg daily SC infusion	Rapid conversion	Inadequate Analgesia.	25:1	Meth SC	Improved analgesia.	Died 30 h after methadone rotation.
8	648 mg	15 mg QID PO	15 mg QID PO	Rapid conversion	Inadequate Analgesia.	10.8:1	Hydromorph PCA IV	No significant improvement in analgesia.	Weaned off methadone. Alive.
9	10,800 mg	36 mg IV infusion over 24 h	600 mg IV infusion over 24 h	Rapid conversion	Myoclonus.	150:1	Meth PCA IV	Less mycolonus.	Died 4 days after methadone rotation.
10	288 mg	1 mg IV NOCTE	10 mg QID SL	Adjuvant. Gradual conversion to methadone	Inadequate analgesia.	N/A	NCA fentanyl IV	Improved analgesia.	Weaning Methadone. Alive.
11	30 mg	0.5 mg QIDPO	2.5 mg TDS PO	Rapid conversion	Inadequate analgesia. Methadone syrup available as elixir.	15:1	PCA hydromorph IV	Improved analgesia. Episode of drowsiness and reduced RR (did not require naloxone).	Able to wean methadone. Alive.
12	1080 mg	1 mg IV NOCTE	10 mg QID SL	Adjuvant for one week, then total conversion to methadone	Inadequate analgesia. Seizures (neurotoxicity) possibly due to hydromorphone.	N/A	NCA hydromorph IV	Improved analgesia. No further seizures.	Weaned off methadone. Alive.
13	Not on regular opioid, but having PRN codeine	2 mg BD PO	4 mg QID PO	Commenced as primary pain management (de novo) at 0.1 mg/kg bd	Complex pain. Neuropathic pain.	Dose based on weight	PRN oxycodone PO	No improvement.	Weaned off methadone. Alive.
14	120 mg	1 mg at night for 1 day and then 3 mg QID SL	10 mg QID SL	Initially adjuvant and then rapid conversion	Neurotoxicity. Severe dysponea. Chest pain. Required elixir.	10:1	PRN morph PO	Less confusion. Improved analgesia and less dyspnoea.	Alive.
15	1068 mg	36 mg IV infusion over 24 h	72 mg IV infusion over 24 h	Rapid conversion	Inadequate Analgesia. Agitation.	15:1	Meth NCA IV	Improved analgesia and sedation.	Died 16 days after methadone rotation.
16	21 mg	0.5 mg TDS PO	0.5 mg TDS PO	Rapid conversion	Available as elixir. Experienced pruritis with morphine.	14:1	PRN meth PO	Stable analgesia.	Weaned methadone. Died 3 weeks after methadone ceased.

Abbreviations: IV, Intravenous; PO, Per Os/Oral; SC, Subcutaneous; SL, Sublingual; NG, Nasogastric; PRN, Pro Re Nata/Medication prescribed on an ‘as-needed’ basis; NCA, Nurse-controlled analgesia; PCA, Patient-controlled analgesia; TDS, Ter Die Sumendum/Medication administered three times per day; QID, Quarter In Due/Medication administered four times per day, Nocte Medication administered at night; Morph, Morphine; Meth, Methadone; Hydromorph, Hydromorphone; N/A, not applicable (where initial methadone dosing was not a rapid conversion from alternate opioid).
